# Preparing for genomic medicine: a real world demonstration of health system change

**DOI:** 10.1038/s41525-017-0017-4

**Published:** 2017-05-01

**Authors:** Clara L. Gaff, Ingrid M. Winship, Susan M. Forrest, David P. Hansen, Julian Clark, Paul M. Waring, Mike South, Andrew H. Sinclair

**Affiliations:** 1 Melbourne Genomics Health Alliance, Victoria, Australia; 2 The University of Melbourne, Victoria, Australia; 3 The Royal Melbourne Hospital, Victoria, Australia; 4 Department of Medicine, The University of Melbourne, Victoria, Australia; 5 Australian Genome Research Facility, Victoria, Australia; 6 Australian e-health Research Centre, CSIRO, Queensland, Australia; 7 Walter and Eliza Hall Institute, Victoria, Australia; 8 The Royal Children’s Hospital, Victoria, Australia; 9 Department of Paediatrics, University of Melbourne, Victoria, Australia; 10 Murdoch Childrens Research Institute, Victoria, Australia

## Abstract

Organisations and governments seeking to implement genomics into clinical practice face numerous challenges across multiple, diverse aspects of the health care system. It is not sufficient to tackle any one aspect in isolation: to create a system that supports genomic medicine, they must be addressed simultaneously. The growing body of global knowledge can guide decision-making, but each jurisdiction or organisation needs a model for genomic (or personalised) medicine that is tailored to its unique context, its priorities and the funds available. Poor decisions could greatly reduce the benefits that could potentially arise from genomic medicine. Demonstration projects enable models to be tested, providing valuable evidence and experience for subsequent implementation. Here, we present the Melbourne Genomics Health Alliance demonstration project as an exemplar of a collaborative, holistic approach to phased implementation of genomics across multiple autonomous institutions. The approach and lessons learned may assist others in determining how best to integrate genomics into their healthcare system.

## Introduction

Genomic technologies have been described as both disruptive and transformative.^1^ The ability to sequence an entire human exome or genome provides vastly more information of potential relevance to a person’s current and future health than was possible previously. As the relationship between sequence variation and disease management becomes better understood, genomic medicine—the use of information from an individual’s genome in the diagnosis and management of their condition—will become increasingly relevant to a broad range of health practitioners. However, numerous barriers to the implementation of genomics into clinical practice exist. These span the realms of workforce (capacity and behaviour change), service design and support, evidence-based practice, reimbursement, e-health (health and biomedical information), and ethics and regulation.^2–4^ Resolving these barriers in order to successfully integrate genomics into routine health care and improve patient outcomes requires change across each health system. Yet, Ginsburg observed that “most health systems around the world are not fit to shepherd genome sciences into routine health care” and that decisions are often made on an ad hoc basis.^5^

The introduction of genomics into a health system is complex, as resolving the diverse barriers requires change across many of its interacting component and health systems are not generally amenable to change.^6^ Several approaches have been taken to address the challenges inherent in genomics. Large scale sequencing initiatives and discovery-based research projects address gaps in understanding the clinical significance of genetic variation.^7, 8^ Global collaborations aim to accelerate progress by fostering the sharing of genomic data.^9^ Increasingly, there is recognition of the importance of implementation research to identify strategies which assist the adoption of genomics by clinicians.^3^ Each effort contributes to an important body of knowledge but forethought and simultaneous change across multiple areas of health care are needed to ensure that a genomic test is available for clinical use (implementation) and that clinicians apply the test and its results in practice (adoption). Focussing predominantly on a single aspect—be it the testing laboratories, technology, a specific medical discipline or clinical problem—will not be sufficient to create the widespread change necessary to ensure that patients’ health is improved by using genomic information in their medical care.

The introduction of genomics will require targeted support and is unlikely to occur by diffusion.^10^ Governments and organisations considering the implementation of genomics need to design a high-level, holistic model for how genomics will be delivered in the future, tailored to their own unique context. Implementing genomics requires considerable investment in infrastructure, personnel, and change management. Getting it wrong is likely to both be extremely expensive and reduce the potential benefits. These risks can be diminished by conducting a demonstration project, whereby a model (e.g., for the delivery of genomics in practice) can be tested to evaluate its strengths and weaknesses.^11^ Importantly, in health care, demonstration projects simultaneously provide an opportunity to foster change and adoption amongst stakeholders, while evidence can be gathered on a potential model’s design, cost and impact. In the case of genomics, demonstration projects provide a framework with potential universal applicability, facilitating local solutions to the challenges faced globally.

The Melbourne Genomics Health Alliance (‘the Alliance’) has conducted a demonstration project as a first step towards integrating genomics into health care across ten independent organisations. The results^12–16^ are now proving indispensable in guiding implementation and adoption of genomics in practice and patient care in Australia. Here we present our experience designing and conducting this collaborative project and share our lessons learned in the hope it will assist others to navigate their own path into the era of genomic medicine.

### The Melbourne Genomics Health Alliance

The Melbourne Genomics Health Alliance (‘Melbourne Genomics’), established as a collaboration in 2013, is tasked with integrating genomics into health care across the Australian State of Victoria—a population of approximately six million people.^17^ Its members are ten leading healthcare, research, academic and service organisations, encompassing five hospitals, five accredited molecular testing laboratories and four clinical genetic services. Patient care across these organisations spans tertiary/ quaternary public hospital services in paediatrics to geriatrics. Public hospital care in Australia is largely funded by the State governments and is available free of charge at the point of care to all residents. There is no provision for reimbursement of genetic or genomic testing through personal medical insurance cover. Limited funding is provided by State and Federal governments, but for many tests no reimbursement is available.^18^ Funding for investigations can be established by application to one of a number of schemes, each with different scope, opportunities and limitations. Currently, health services have access to funding for only a limited number of clinically indicated genomic sequencing tests, restricting substantive testing to those eligible for research studies or able to pay themselves.

The members of the alliance recognised the need to work together to optimise the use of genomic information in clinical practice and research across the Victorian State health care system. To succeed in this goal, clinicians, patients, clinical laboratory scientists and researchers need seamless, secure access to genomic data and information. The members agreed to establish a common genomic data management platform that enables the analysis, storage and use of clinically generated genomic data from multiple diagnostic laboratories and its availability at six health care organisations and to several research institutions. This is an unprecedented level of change and cooperation between independent hospitals, accredited testing laboratories and academia—all organisations having separate and unique governance structures.

Importantly, the Alliance recognises an individual organisation’s right to make their own business decisions (e.g., to provide an accredited testing laboratory service) while supporting collaboration to drive change and optimise the use of resources to improve patient care and research opportunity. This paper focusses on the areas of change that were addressed collaboratively, not those that were within the remit of individual organisations (e.g., accreditation of testing).

### Melbourne Genomics demonstration project

Demonstration projects sit at the interface between policy, research and implementation. They test the practical application of knowledge or experience—such as a structural innovation (e.g,. technology) or non-structural innovation (e.g., health programme or policy)—in a chosen setting, preferably under real conditions. They are usually conducted for a limited time and in effect test a prototype or a ‘proof of concept’ model to provide information, but are not intended to be scaled up. By contrast, pilot projects are designed and delivered as a programme is expected to be conducted, and can be scaled up for full implementation.^19^ Evaluation determines a model’s feasibility (can the model be built?), and requirements (what does the model need to do?) and/or impact (can it make a difference?).

A demonstration project which can guide the broad implementation and adoption of genomics in a healthcare system needs to resolve the numerous barriers that occur across the whole health care system. It should provide evidence of clinical utility and cost-effectiveness to inform test reimbursement, clinical guidelines and medical decision-making. It needs to determine how genomic data can be ethically and robustly managed so the information is available across institutions for clinical care and research. It also provides an opportunity to foster change in clinical and organisational behaviours such that genomic information is incorporated in the practice of medical specialists.

**Fig. 1:**
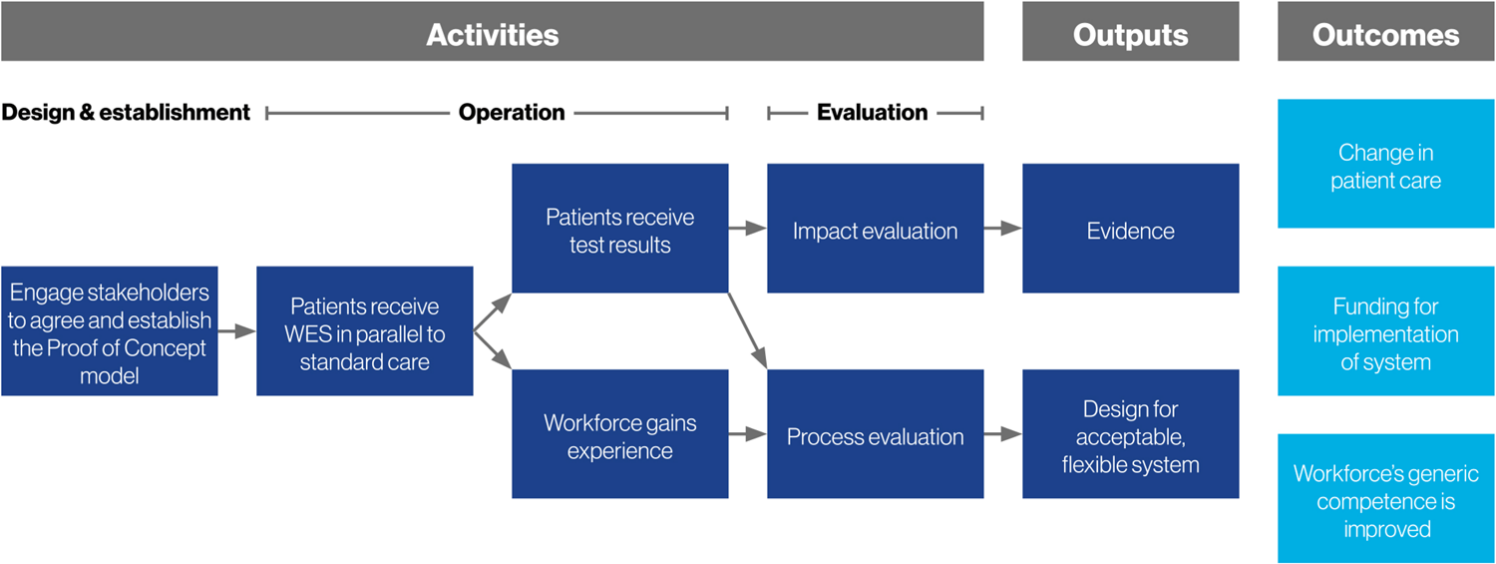
Demonstration programme logic. This diagram summarises the programme logic model, that is the relationship between the demonstration project’s activities, its outputs and the intended outcomes

The Melbourne Genomics demonstration project was designed to provide evidence for the cost-effective use of genomics in clinical care, identify acceptable and practical clinical and diagnostic information systems, policies and procedures, and upskill the workforce through hands-on experience. The chain of actions and intended outcomes (known as the ‘programme logic’^20^) of the demonstration project is shown in Fig. 1.

**Fig. 2:**
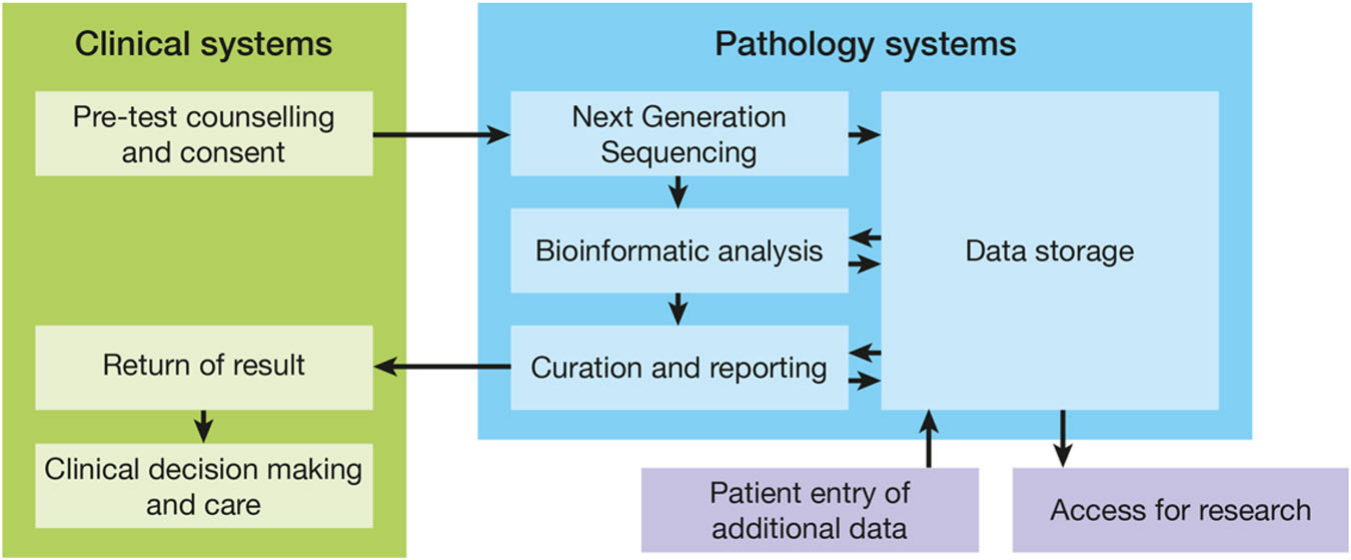
Steps in the pathway for patient testing and use of the genomic data undertaken in the demonstration project

Conducting the demonstration project entailed establishing a ‘proof of concept model’ comprising governance, policies, procedures, infrastructure, and software applications (Table 1). Each component of the model was developed and agreed through a consensus decision-making process. Decisions were guided by placing the principles which underpin clinical practice ahead of research, technological or commercial imperatives and political drivers. Each aspect of the proof of concept model was evolved and evaluated within health care delivery (clinical and pathology services) by offering genomic sequencing to patients meeting specific criteria (see Patient Testing below). The broad steps involved in providing testing and using genomic data within the demonstration project are shown in Fig. 2.

**Table 1:** The demonstration project's ‘proof of concept model’

Step	Policies and guidelines	Agreements and forms	Information management systems and infrastructure (software or provider)
Pre-test counselling and consent	Genetic counselling guidelines Secondary findings policy	Consent form	N/A
Clinical data entry	Standard terminologies where available	N/A	Phenotips^a^
Next Generation Sequencing	Sample quality standards	Sample metadata file	N/A (members own)
	Sequencing data quality standards		
Bioinformatics analysis	Analysis standards	Operating manual	Modular bioinformatic pipeline (C-pipe)^1^
	Reanalysis for clinical purpose		High performance computational services (VLSCI)
Curation and reporting	Variant classification and prioritisation schema	Template report for test results	Variant curation tool and database (MG-LOVD)
	Guidelines for multidisciplinary review		
Return of result to patient	Verification and return of results policy	N/A	N/A
Clinical decision-making and care	None (clinician’s own decision)	N/A	N/A
Data storage	N/A^b^	N/A	Storage area network (VLSCI)
Access for research	Access for research policy and procedure	Data access agreement Consent form (as per counselling and consent)	Research storage service with time-limited email link for access (RDSI)
Federation to electronic health data	As per BioGrid Platform	BioGrid member agreement	BioGrid
Patient entry of additional data	N/A	Minimum data set	Patient entered data tool Data linkage (BioGrid)

The table shows the policies, guidelines, agreements, forms and systems for information management which form the prototype model tested in the demonstration project. Further detail on the information management systems and infrastructure is given in the Supplementary material.

*MG-LOVD* Melbourne Genomics modified Leiden Open Variant Database, *RDSI* Research Data Storage Initiative, *VLSCI* Victorian Life Sciences Computational Initiative

^a^ Childhood syndromes patients only

^b^ policies for data storage were as required by the National Statement for Ethical Conduct in Human Research

Four broad groups of activities were central to the demonstration project: patient testing, evaluation, workforce development and e-health.

#### Patient testing

Clinicians offered patients whole exome sequencing in a routine clinic setting prospectively, in parallel with standard clinical investigations. Therefore, patients were eligible if they had not had previous molecular investigations. This design allows evaluation of genomic sequencing as an early or first tier investigation and complements retrospective studies of patients who have undergone considerable investigation before receiving a diagnosis.^8, 21, 22^ Analysis and reporting of variants were restricted to genes currently known to cause the relevant condition or affect its treatment. Patients with one of five diverse clinical indications (Table 2) were offered participation in the demonstration project, with pre- and post-test genetic counselling. The clinical conditions span adult and paediatric care; germline and somatic testing; new and well characterised genes; those with well-established molecular testing pathways and those without. The diversity of these ‘flagship conditions’ enabled us to evaluate when common procedures and systems can be used and when they must be refined for specific diseases, as well as the impact of providing genomic sequencing for different indications.

**Table 2:** Description of the Flagships

Name of ‘Flagship condition’	Description	Test type	Number of genes	Number tested	Clinical disciplines
Childhood syndromes	Children (average age 2.5 years) with features suggestive of a single gene disorder.	Germline	2820	142	Medical genetics^a^
					Neonatology
					Paediatrics
Inherited neuropathies	Adults, adolescents and children with a clinical diagnosis of Charcot–Marie–Tooth disease, a group of inherited neuropathies with a broad range of phenotypes, inheritance patterns and causative genes.	Germline	55	50 (25 paediatric 25 adult)	Paediatric neurology^a^
					Adult neurology
					Medical genetics
hCRC	Adults with personal and/or family history meeting criteria for inherited syndromes causing colorectal cancer.	Germline	17	35	Gastroenterology^a^
					Medical genetics
					Oncology
Focal epilepsy	Adults, adolescents and children with focal epilepsy in the absence of a structural brain lesion or past history suggestive of previous brain insult	Germline	59	41 (12 paediatric 29 adult)	Adult neurology^a^
					Paediatric neurology
					Medical genetics
AML	Patients with AML aged 70 or younger where the clinician considers that additional genomic testing may assist in understanding prognosis and/or contribute information for future treatment decisions.	Somatic	12	45 (11 paediatric, 34 adult)	Adult haematology^a^ Paediatric oncology

*hCRC* hereditary colorectal cancer, *AML* acute myeloid leukaemia

^a^ Clinical discipline of the flagship leader

#### Evaluation: evidence for application

To inform decision-making about how genomics is best implemented into health care, rigorous evaluation is needed. As with genetic services,^23^ the use of genomic sequencing in practice can be considered a ‘complex intervention’ by the Medical Research Council (MRC) definition^24^ as more than one group is affected (e.g., the patient and their blood relatives), there are numerous, varied outcomes, and its use requires some degree of tailoring. Complex interventions are commonly tested using experimental designs to determine the efficacy of an intervention. However, the structured nature of experimental designs does not allow insights into how adoption of the intervention can be supported. We applied a hybrid effectiveness—implementation design,^25^ which is better suited to demonstration projects. This design allowed us to identify when genomics is a cost-effective investigation (impact evaluation), as well as how it can best be offered (process evaluation), alongside activities designed to foster implementation (Fig. 1).

By providing genomic sequencing in parallel to usual care, we were able to directly and prospectively compare the rate of detection of mutations, the impact on patient care (i.e., the number of patients whose care changed in response to the test results and the nature of this change) and cost effectiveness of these two clinical pathways. In contrast to retrospective studies—where patients diagnosed through usual care are not considered in calculations of detection rate or cost effectiveness—this approach best reflects the ‘new world’ of genomic medicine where clinicians can order genomic sequencing at first presentation. Consequently, we found a diagnostic rate higher than 50% with genomic sequencing for children with features strongly suggestive of monogenic conditions (‘Childhood Syndromes’) and one third of those diagnosed receiving a change in care as a result.^12, 14^ This is consistent with other clinically ascertained infant cohorts in the US and Canada.^26–28^ Our work provides evidence to guide the timing of genomic sequencing relative to other investigations. For example, we have demonstrated that if whole exome sequencing was applied after exhaustive investigations of children under 2 years of age with features strongly suggestive of monogenic conditions, every additional diagnosis made by whole exome sequencing would cost US$6327. Strikingly, when whole exome sequencing could replace most investigations there was a saving per additional diagnosis of US$1702.^13^ Evaluation of this rigour is essential to support cases for funding and reimbursement. One avenue to obtain federally subsidised testing is through the Medicare Benefits Scheme^29^ and a submission for inclusion on this scheme has been made based on our data.

Equally importantly, this design provides evidence for when such testing is not warranted. Although whole exome sequencing yielded a diagnostic rate of 25% in patients investigated for hereditary colorectal cancer (hCRC), we found no improvement in comparison to usual care, as all patients standardly received a panel test (unpublished data). Our experience demonstrates the importance of gathering evidence for each specific clinical indication and consideration of health and health economic outcomes as well as diagnostic rate. Although the diagnostic yield for patients with focal epilepsy was relatively low (12.5%), one patient’s management and clinical outcome was profoundly improved.^16^ In contrast, 40% of patients with hereditary neuropathy gained a diagnosis^15^ but immediate changes in patient management as a result were limited (unpublished data).

As important as outcomes is the efficiency of the processes. In order to identify acceptable and practical policies and procedures, data was gathered from participating patients and the clinicians, diagnostic scientists and informaticians involved in the demonstration project. As well as enabling iterative improvements, the process evaluation has provided rich information about key pressure points, laying the basis for further implementation research and identification of systems which will support the use of genomics in clinical service delivery. These results will be reported separately.

#### Workforce development: fostering clinical adoption

Medical practitioners are concerned about being adequately prepared for the use of genomics in their practice. Christiansen and colleagues reported that contributing factors include a lack of knowledge and laboratory guidance, time pressures, and a lack of standards.^30^ While continuing medical education is a pathway for practitioners to overcome knowledge deficits,^31^ education alone is unlikely to be sufficient to facilitate appropriate use of genome-scale testing by medical specialists.^32^ Behaviours such as clinical decision-making are affected by an individual’s capability, opportunity and motivation to act;^33^ which are in turn influenced by both factors intrinsic to the practitioner and external factors such as service environment.^34^

Our strategy for fostering change was centred on the use of experiential learning, providing opportunity with the intention of building capability and motivation. The outcomes we sought were multifaceted: to build clinicians’ understanding of the complexities of genomic sequencing, to foster future adoption of evidence regarding the value of incorporating genomics by clinical disciplines, and to ensure that the systems and processes fully implemented in the future will be embraced by clinicians. Clinicians from ten clinical specialisations (Table 2) had the opportunity to gain “hands-on” experience of genomic sequencing in practice through patient testing. As well as managing testing and test results, they participated in multidisciplinary meetings to review results and determine clinical significance. Clinicians were responsible for the activities relating to patient testing for each condition, including the evaluation of impact. At the outset, clinicians were encouraged to engage in all decisions relating to design of the demonstration model and, as described above, were key informants in its evaluation. We found surprisingly few challenges to engaging clinicians in the various capacity building activities. Good clinical leadership assisted and the main barrier was logistics, for example scheduling result review meetings that suited clinicians from different hospitals. Anecdotally, any variation in engagement between medical speciality groups appeared to relate more to clinical utility of the testing than medical discipline.

The clinicians who participated in these activities work in tertiary and quaternary clinical settings, as this is where the immediate need for genomics is currently concentrated. Our strategy for fostering adoption is not scale-able to clinicians across the entire workforce, but it does yield a cohort of informed clinicians to lead change within their disciplines as testing becomes more widespread. Demonstration projects designed for adoption of genomics in secondary and primary care may be beneficial when genomic sequencing tests become available to practitioners in these settings.

#### E-health

A fundamental difference between whole genome/exome sequencing and testing a limited number of genes is the potential for reanalysis of the data in the future. Ideally, a person’s genomic information should be available to themselves and their health practitioners over their life course, enabling the underlying data to be re-analysed as analytic tools and evidence for its use in care improves. In fact, the American College of Medical Genetics and Genomics recommend that periodic re-evaluation of an individual’s genome is desirable.^35^

**Fig. 3:**
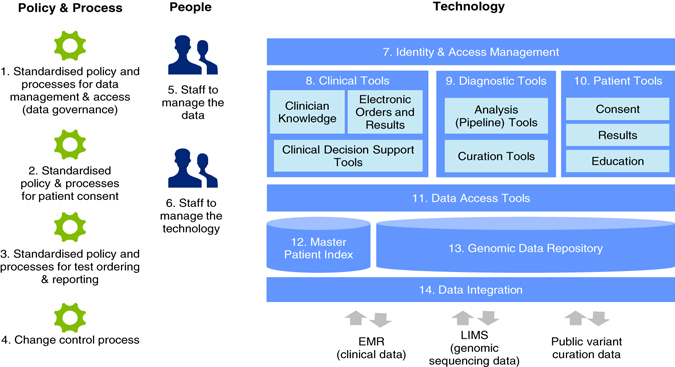
Conceptual diagram of the Melbourne Genomics Health Alliance shared information management platform for future implementation. The core of the common genomic data management platform is the Genomic Data Repository—a central place to store genomic sequence data. The Clinical Tools, Diagnostic Tools and Patient Tools are shared by the Alliance members and integrate with their own systems including EMR and LIMS. Further detail is in Supplementary Material 1

Establishing a platform, which enables the efficient management and use of clinically generated genomic information, however, is far from a simple task.^36, 37^ To avoid duplication, the members agreed to establish a single interconnected platform, which would provide functionality for laboratory genome analysis and systems, guide clinician decision-making at the point of ordering tests and interpreting the results, integrate with each organisation’s separate information systems, and support patient access to genomic information. Figure 3 shows the components of a shared platform for management of genomic data and information and its relationships with the data management systems of the members. Crucially, there needs to be a two-way flow of information between clinicians and laboratories, via the genomics platform and the usual clinical and laboratory information management systems (LIMSs) within each member organisation. Of course, the platform needs to be compliant with privacy and health data standards and have strong data governance—that is, clear definition of the way in which data is managed to ensure its availability, usability, integrity, and security. More information on the components are provided in Supplementary Material 1.

The feasibility of this ambitious objective was tested by creating a proof of concept information management platform used by all laboratories for the demonstration project (Table 1 and Supplementary Material). The functionality of this was limited to the infrastructure and software applications necessary for bioinformatics analysis^38^ curation of variants and reporting, and storage of data relevant for reanalysis and research. Electronic medical records (EMRs) had not been implemented within the participating hospitals at the time, so integration could not be tested. Instead, the proof of concept variant curation database was linked with a federated health data platform, BioGrid (BioGrid.org.au). Akin to fostering change amongst clinicians, participation in this aspect of the demonstration project enabled clinical laboratory scientists to gain first-hand experience in using applications that provide genomic information, prior to providing whole exome sequencing as a diagnostic test. A myriad of ethical, legal and technical issues required resolution for laboratory analysis and making access to genomic data available to all researchers within the Alliance. This experience was invaluable, enabling better informed decisions to be made about the functionality needed for the long-term systems to be implemented. The detailed system design of the long-term systems was based on this work and undertaken after completion of the demonstration project. Further information is available from the corresponding author.

### Challenges and lessons learnt

#### Leadership

The Melbourne Genomics Health Alliance has become a paradigm for successful collaboration in Australia. A key success factor was the role of the executives of each member organisation. The Alliance and its demonstration project was initiated by the executive leader of each of the founding Alliance member organisations. It was these leaders who agreed the vision and objectives, provided funding, and set expectations for stakeholders within their organisations. They clearly signalled that this was to be strongly collaborative in practice as well as intent when they funded the demonstration project equally, irrespective of the organisation’s turnover or scale of genomics activity. This message was clear to those in their organisations, though stakeholders appeared to be primarily motivated by the desire to ensure genomic sequencing was available to patients. There were no direct incentives (in the form of Key Performance Indicators, for example) placed on stakeholders within each organisation to succeed.

#### Collaboration and engagement

Earlier efforts by the Alliance members to collaborate at an organisational level had failed, in part because agreement could not be reached on how existing activities could be aligned. As a result, some stakeholders had concerns about hidden agendas, for example the consolidation of diagnostic laboratories. To ensure that the Alliance was a “coalition of the willing” it was explicitly stated that consolidation of laboratories was not an objective. Instead, a vision was developed for how genomics would be delivered in 5 years’ time (rather than by whom). This allowed stakeholders to make business decisions which aligned with the 5-year vision of their own volition without compromising their current work. The accredited laboratories are an exemplar of how this worked in reality. Each laboratory decided independently the extent to which they would continue to develop gene testing panels and/or put resources into exome sequencing. They also determined the extent of their involvement in generating the patient’s sequence and in interpreting and reporting the results for the demonstration project. Convergence between the members’ individual strategic development and the 5 year vision was actively fostered by ensuring that the governance of the demonstration project included stakeholders at a range of levels within each member’s organisational structure. This convergence was gradual and determined independently within each of the relevant organisations.

Multidisciplinary collaboration on the demonstration project greatly strengthened the outcomes. Stakeholders engaged were those who needed to incorporate genomics into their role (clinicians, diagnostic scientists), those with experience of genomic sequencing (laboratory researchers, bioinformaticians), those who would receive testing (patients, community advisors), evaluators and those with other technical expertise (health informaticians, software and database developers). Obtaining consensus within multidisciplinary groups was challenging; clinical care, diagnostics, information management and research have different and at times conflicting drivers. Institutional allegiances at various levels added another degree of complexity. However, we adopted a number of strategies to promote the achievement of consensus and this proved easier than anticipated. Firstly, the principles or criteria upon which a decision would be made were agreed before any options were considered. Table 3 lists the overarching principles that guided the programme. These were elaborated as relevant for individual decisions, such as the selection of a bioinformatic analysis platform. The focus on patient care provided little opportunity for the pursuit of self interest and the use of guiding principles promoted clarity and transparency of decision-making. Secondly, in general, we found the people closest to the “coal face” were best placed to make the assessment of the options against the set criteria and tended not to be parochial. Lastly, people seemed more willing to reach agreement on decisions where the outcome—a policy, guideline or software—would be subject to evaluation than they may have been for decisions with longer-lasting consequences.

**Table 3:** Principles for decision-making

Overarching principles
*Clinically driven*
• Patient participation and autonomy will be fostered.
• Genome data will be analysed according to clinical indication.
*Collaborative*
• Genomic data will be shared for clinical and research use according to existing regulations for health records and ethical standards.
• Common systems and standards apply where they will facilitate access to and (re-) use of genomic information by multiple partners throughout the patient’s lifetime and across the research-translation continuum.
• Each member remains responsible for business decisions about the services it provides and the resourcing and quality assurance of those services.
• Each hospital remains responsible for procurement of genomic testing for their patients, subject to the provider meeting the shared standards.
*Sustainable*
• Decisions during development will be ‘user-focussed’—where those users are variously patients, clinicians, researchers and diagnostic laboratories—and evidence-based.
• Systems will be designed for optimal (financial) sustainability, scalability, incorporation of future -omics advances and future inclusion of organisations outside the founding members.

#### Agility and project management

Generally, clinicians and researchers were very familiar with the process of research, where the work to be undertaken to address a hypothesis and the allocation of funds to achieve this have been detailed in advance, in a successful grant application. In contrast, the demonstration project was funded as an implementation project. The methodology was more akin to an information technology implementation project, with the member organisations being the project sponsors (funders), the agreed scope of work being documented in a project brief and procedures and systems being developed agilely. This was initially difficult for stakeholders to understand. Some voiced concern that there was not a detailed end-to-end description of every step, while others were anxious that decisions had already been made without their input. Good governance, inclusiveness and transparency of decision-making were crucial in allaying these concerns. Another source of confusion was the necessary delineation between areas of work that were identified as collaborative and those that remained fully in the realm of the individual organisation (e.g., sequencing technology acquisition).

In order to build capability and activity within the Alliance organisations, all parts of the demonstration system were built and run by stakeholders, incorporating external expertise as required. To reduce the risk of not completing the demonstration project, a small, dedicated project team facilitated the collaborative process, and were responsible for ensuring that decision-making aligned with the objectives of the Alliance. The project team also assisted management of stakeholders’ competing demands on their time, ensuring rapid progress. However, in the context of a multi-institutional demonstration project, a project team can inadvertently reduce stakeholders’ sense of ownership, jeopardising future adoption. The members and stakeholders are the Alliance, not the project team: stakeholders and programme team all needed to be reminded of this at times.

In retrospect, our decision to focus resources on project delivery, without allocating more resources to communication within the Alliance, was a mistake. The use of a demonstration project was a radical paradigm shift for stakeholders’ and better and broader communication at the outset may have lessened some of the early confusion. Time and exposure may also have been necessary for stakeholders to fully engage.

## Conclusion

We have shown that the framework of a demonstration project is well suited to testing a model for the application of genomics in clinical care and can build substantial momentum towards implementing genomics into our healthcare system. The conduct of the demonstration project itself fostered change across many disciplines, domains and organisations. As the complexities were even greater than envisaged at all levels and in all activities, it has created a strong foundation for the next phase of work—implementation of systems into the health systems. The State Government of Victoria and members have provided further funding (AUS$35M) to implement genomics over the next 4 years, based on evidence from the demonstration project. Melbourne Genomics subsequently became the blueprint for the development of a national initiative, namely: The Australian Genomics Health Alliance funded (AUD $25M) by the Federal Government’s National Health and MRC.

The proof of concept model we developed and tested may provide some usable insights and our suggestions for others are provided in Table 4. In 2013 Manolio and colleagues proposed a roadmap for the implementation at a single institution.^39^ Most of the steps they proposed were very pertinent to the path the Melbourne Genomics Health Alliance took, but the order of these steps was quite different. These differences were largely due to the ‘top down’ initiation of the work in Melbourne and both the scale (implementation across multiple institutions) and the multi-faceted nature of our programme.

**Table 4:** Suggestions for those considering a collaborative demonstration project in genomics

Suggestion	Illustration from the demonstration project
Collaboration and agreement	
Secure support from the participating institutions leadership	The high level, strategic view of what was to be achieved was determined by the members’ executive leader. Financial contribution (AUD$250,000 per annum per institution) ensured that organisations were committed to succeeding.
Build trust by choosing a host institution that is perceived by all members neutral and enabling.	The host institution does not provide clinical care or conduct diagnostic testing, but does have procurement processes that supported rapid progress.
Consider which governance structure bests supports both implementation within the participating organisations and future sustainability, e.g a collaboration agreement or company structure	A collaboration agreement was chosen in order to better retain the members’ and stakeholders’ sense of ownership of the activities and minimise the administration required.
Appoint an independent chairperson for the project steering committee.	The independent chair actively fostered a collaborative culture in this committee and was a powerful voice externally.
Engage with representative users, from multiple institutions, across the entire investigation cycle	The clinicians and medical scientists from the collecting laboratories and the labs performing the tests, were involved in determining the workflows and were interviewed for the process evaluation.
Where feasible, ensure all organisations are contributing to the activities they wish to be involved in and don’t force anyone to participate	One laboratory decided to only sequence patients for the demonstration project, another to only curate and report results and a third provided end-to-end testing.
Design and project conduct	
To ensure early successes and build momentum, consider using agile development approaches	The bioinformatic pipeline, for instance, was selected and operational within 6 months
Conduct activities in the context in which genomics will take place in the future and as close to the expected practice or protocol care as possible	Testing was performed by accredited laboratories, not research laboratories.
	Testing was undertaken in batches as they arrived at the laboratory, not as a cohort or grouped by clinical indication.
Engage users with varying levels of knowledge and expertise in genomic medicine step-by-step when planning an implementation. A system designed around only the most expert users may not work well in the real world.	The initial result report template was developed at a workshop which included geneticists, other medical disciplines and a community representative. The detail advocated by specialist geneticists was initially overwhelming for clinicians without experience in genomics.
Construct the project management team to include both experienced project managers and subject matter experts –	The project management team were largely subject matter experts, who understood the technical task at hand, but insufficient experienced project managers.
Consider competitive processes to determine which clinical indications will be tested.	We used a consensus approach due to time constraints. Subsequently we find a competitive process results in greater trust in the process and motivation by the participating clinicians.
Be prepared for varying levels of information technology sophistication between differing health services.	Research infrastructure provided an environment that allowed rapid deployment and nimble testing of proof of concept bioinformatic analysis, variant curation and research access tools. Distinction needed to be drawn between research drivers (which require novel, cutting edge approaches and flexibility) and the requirements of clinical systesms (accuracy, reliability and reproducibility),
Establish good natured competition between clinical groups to accelerate recruitment.	The number of patient tests available for each clinical indication was contingent on a half-way review of progress. Recruitment progress was circulated fortnightly.
Allow more time for every activity than you expect it will need, as it will be more complex than you expect.	A 1 year programme took 2 years to complete, with the delays largely due to recruitment, testing turn around time, and availability of data for evaluation (e.g cost data)
Outcomes	
Measure benefits at two levels: (1) the benefits arising from genomic sequencing as evidence for future value and (2) the benefits arising from conduct of the demonstration project to determine the impact of the investment made by the funders.	(1) Evidence for the use of genomics: Cost effectiveness of exomes in comparison to usual care
	(2) Benefits from the demonstration project: funding from the State Government.
Support a clinician to conduct the evaluation of the impact of genomic sequencing.	The evaluations were most thorough when a clinician was highly motivated (e.g undertaking the work as part of a PhD) as we did not fund clinicians. Clinicians are now funded to coordinate activity for each clinical indication and conduct the evaluation.
Think broadly about the potential issues in implementation in the laboratory and clinic at the outset.	Consideration should have been given to how the design of the demonstration project could provide data for the accreditation process.
When evaluating process, focus on those that are relevant to practice	When interviewed, many stakeholders identified issues that related to the research study (e.g. research consent requirements) and not clinical care.

The differing histories, drivers and health care systems mean that other organisations and countries are likely to have a different view of how genomics can be best implemented. These differences serve to emphasise the value of demonstration projects as a universally applicable framework for identifying how complex innovations such as genomics can most successfully be implemented in health care systems.

## Change History

A correction to this article has been published and is linked from the HTML version of this article.

## Electronic supplementary material

Supplementary Material
