# Erratum: Preparing for genomic medicine: a real world demonstration of health system change

**DOI:** 10.1038/s41525-017-0028-1

**Published:** 2017-10-05

**Authors:** Clara L. Gaff, Ingrid M. Winship, Susan M. Forrest, David P. Hansen, Julian Clark, Paul M. Waring, Mike South, Andrew H. Sinclair

**Affiliations:** 1 Melbourne Genomics Health Alliance, Victoria, Australia; 2 The University of Melbourne, Victoria, Australia; 3 The Royal Melbourne Hospital, Victoria, Australia; 4 Department of Medicine, The University of Melbourne, Victoria, Australia; 5 Australian Genome Research Facility, Victoria, Australia; 6 Australian e-health Research Centre, CSIRO, Queensland, Australia; 7 Walter and Eliza Hall Institute, Victoria, Australia; 8 The Royal Children’s Hospital, Victoria, Australia; 9 Department of Paediatrics, University of Melbourne, Victoria, Australia; 10 Murdoch Childrens Research Institute, Victoria, Australia

**Erratum to**: *npj Genomic Medicine* (2017); doi:10.1038/s41525-017-0017-4; Published 01 May 2017

A citation was inadvertently deleted. Reference 13 should be cited at the end of the sentence on page 16, column 2, paragraph 1 that reads:

“Strikingly, when whole exome sequencing could replace most investigations there was a saving per additional diagnosis of US$1702.^13^”

This error has now been corrected in the HTML and PDF versions of this article.

