# Supplementary material for: Preparing for genomic medicine: a real world demonstration of health system change
Source: NPJ Genom Med. 2017 May 1;2:16. doi: 10.1038/s41525-017-0017-4 (PMC5677913; doi:10.1038/s41525-017-0017-4)
Supplement: Supplementary file 1 — Supplementary Material [file 41525_2017_17_MOESM1_ESM.docx]

Supplementary Material

Components of the Shared Information Management Platform shown in Figure 3 and ‘proof of concept’ tools used in the demonstration project.

| **Platform**  **Component** | **Sub component** | **Description** | **Information management system for Demonstration project** |
| --- | --- | --- | --- |
| Identity & Access Management |  | Systems to ensure that the right people get the right access to the right tools and data, ensuring privacy and security are maintained | Inbuilt within each tool (e.g. variant curation tool and research data access tool) requiring separate sign-in. |
| Clinical tools | Clinician knowledge and Clinical decision support | Tools (e.g. knowledge bases) to enhance clinical decision-making and productivity when ordering tests and applying results in care integrated within the hospital ordering and Electronic Medical Record software. | None |
| 8. Clinical tools: | electronic orders and results | Test ordering will be implemented into existing pathology ordering systems.  Reporting: | Ordering: manual systems as no electronic test ordering was available in the members. The study database automatically created the meta data file which provided the information required to perform analysis and curation. For Childhood Syndromes HPO terms were captured using the Phenotips software.  Reporting: result reports were generated by the Variant Curation database and manually modified as required. |
| Diagnostic Tools: | Analysis pipeline tools | A commercial platform supporting bioinformatic analysis using multiple pipelines as required clinically. | The “C-pipe^1^ bioinformatics pipeline hosted on the high performance computational services at one of the members, VLSCI. |
| Diagnostic tools | Curation tools | A commercial tool to support the classification, interpretation and reporting of variants licensed for use across the Alliance. | An open source software solution, Leiden Open Variant Database, customised by alliance members to enable classification, interpretation and reporting of variants across multiple sites. |
| Patient Tools | Consent, Results and Education | Content and data capture related to genomics will be provided through patient portals being implemented as part of hospital EMR programs. | A portal for patient data entry (Cart-Wheel.org) was modified and trialed.  Patient feedback on online patient information and hard copy materials obtained |
| Data Access Tools |  | Availability of data in the genome data repository for secondary uses such as research. | Web-based tool developed for online request for access to data  Access provided through VicNode storage service using a time-limited email link for access) (vicnode.org.au) |
| Master Patient Index |  | Issues a unique identifier for the patient to ensure their personal details do not need to be used and identifies and resolves duplicates. The Victorian State Wide Master Patient Index currently being developed by the Department of Health. | Not applicable. |
| Genomic Data Repository |  | A data repository that will catalogue and, store sequence data and enable searching and retrieval for clinical re-use and research. | Data storage within the variant curation tool and file storage in a secure environment that allowed data access |
| Data Integration |  | Provides the infrastructure for connecting users and systems to the relevant information via defined interfaces | The BioGrid service, a Victorian wide data sharing service, was used to access data about patients from other data sets. |

1 Sadedin, S. P. *et al.* Cpipe: a shared variant detection pipeline designed for diagnostic settings. *Genome medicine* **7**, 1-10, doi:10.1186/s13073-015-0191-x (2015).
